# Frequency-Tagging Electroencephalography of Superimposed Social and Non-Social Visual Stimulation Streams Reveals Reduced Saliency of Faces in Autism Spectrum Disorder

**DOI:** 10.3389/fpsyt.2020.00332

**Published:** 2020-04-28

**Authors:** Sofie Vettori, Milena Dzhelyova, Stephanie Van der Donck, Corentin Jacques, Jean Steyaert, Bruno Rossion, Bart Boets

**Affiliations:** ^1^Center for Developmental Psychiatry, Department of Neurosciences, KU Leuven, Leuven, Belgium; ^2^Leuven Autism Research (LAuRes), KU Leuven, Leuven, Belgium; ^3^Institute of Research in Psychological Science, Institute of Neuroscience, University of Louvain, Louvain-La-Neuve, Belgium; ^4^Université de Lorraine, CNRS, CRAN-UMR 7039, Nancy, France; ^5^Université de Lorraine, CHRU-Service de Neurologie, Nancy, France

**Keywords:** frequency tagging, autism spectrum disorder (ASD), EEG, social attention, faces

## Abstract

Individuals with autism spectrum disorder (ASD) have difficulties with social communication and interaction. The social motivation hypothesis states that a reduced interest in social stimuli may partly underlie these difficulties. Thus far, however, it has been challenging to quantify individual differences in social orientation and interest, and to pinpoint the neural underpinnings of it. In this study, we tested the neural sensitivity for social versus non-social information in 21 boys with ASD (8-12 years old) and 21 typically developing (TD) control boys, matched for age and IQ, while children were engaged in an orthogonal task. We recorded electroencephalography (EEG) during fast periodic visual stimulation (FPVS) of social versus non-social stimuli to obtain an objective implicit neural measure of relative social bias. Streams of variable images of faces and houses were superimposed, and each stream of stimuli was tagged with a particular presentation rate (i.e., 6 and 7.5 Hz or *vice versa*). This frequency-tagging method allows disentangling the respective neural responses evoked by the different streams of stimuli. Moreover, by using superimposed stimuli, we controlled for possible effects of preferential looking, spatial attention, and disengagement. Based on four trials of 60 s, we observed a significant three-way interaction. In the control group, the frequency-tagged neural responses to faces were larger than those to houses, especially in lateral occipito-temporal channels, while the responses to houses were larger over medial occipital channels. In the ASD group, however, faces and houses did not elicit significantly different neural responses in any of the regions. Given the short recording time of the frequency-tagging paradigm with multiple simultaneous inputs and the robustness of the individual responses, the method could be used as a sensitive marker of social preference in a wide range of populations, including younger and challenging populations.

## Introduction

Individuals with autism spectrum disorder (ASD) are characterized by impairments in social communication and interaction, and the presence of restricted and repetitive patterns of interests and behavior. They often struggle with social interactions in daily life (1). Several developmental accounts [e.g., (2–4)] propose a developmental cascade in which early-onset impairments in social attention deprive children of adequate social learning experiences necessary for the development of successful social interactions (5). As a result, the classical preference for social over non-social stimuli (e.g., faces over artefacts) that is observed in early life and throughout development [e.g., (6–9)] might not arise, further disrupting the development of social skills and social cognition, and ultimately social functioning and interaction. Due to differences in neural reward processing, autistic people may not experience social stimuli as rewarding as neurotypical people do [e.g., (10–12)]. However, findings on this matter have not been entirely consistent. While Zeeland et al. (12), find that the response to social rewards is particularly decreased in children with ASD in relation to social reciprocity, reward responses to non-social stimuli were also reduced. Therefore, whether aberrant reward processing in ASD is confined to social stimuli or reflects a more general deficit in stimulus-reward associations remains unclear. Likewise, whether attentional processing is particularly impaired for social stimuli or for more complex stimuli in general, remains inconclusive (13).

Empirical evidence from eye-tracking studies confirms that the classical attentional preference for social versus non-social stimuli in the general population is reduced or even absent in individuals with ASD. While evidence is mixed during the first months of life, infants who later develop autism symptoms show reduced social orienting by the end of the first year (14, 15). Recently, a large cohort study (16) with toddlers (12–48 months old) reported enhanced preference for visual stimuli displaying geometric repetition as compared to social stimuli (e.g., videos of playing children) in children later diagnosed with ASD, in particular for an ASD subtype with more severe symptoms. These results suggest that perhaps, the decreased social engagement observed by the end of the first year of life is the developmental *consequence* of impairments in a different functional system during infancy. Hence, an alternative hypothesis is that decreased social orienting and motivation could, for example, be a consequence of difficulties in processing the incoming social information, rather than their cause (14, 15).

In a meta-analysis, Frazier and colleagues (17) analyzed and integrated results of 122 independent studies investigating gaze patterns in infants, children, and adults with ASD as compared to TD individuals. They concluded that individuals with ASD show a basic difficulty selecting socially relevant versus socially irrelevant information. Moreover, gaze abnormalities persist across age and worsen during the perception of human interactions. Other meta-analyses of eye-tracking studies report similar evidence for decreased visual attention to social stimuli in individuals with ASD (18, 19), and demonstrate that an increase in social load, either by including child directed speech or by including several persons interacting with each other, further results in decreased attention to social stimuli in participants with ASD. Thus, generally, eye-tracking research supports a reduced preferential looking bias for social stimuli in ASD. However, effect sizes are moderate and vary across studies, stimuli, and designs (18, 19).

Eye-tracking, often the methodology of choice to study social preference, conveys information about overt orienting processes. However, covert attention is not assessed by eye-tracking studies, possibly resulting in an underestimation of the social bias in studies comparing individuals with and without ASD. The covert processing of social information in ASD has been mostly studied *via* event-related potentials (ERPs) extracted from electroencephalography (EEG) [e.g., (20–26)]. The vast majority of studies focused on the N170, a negative ERP peaking at about 170 ms over occipito-temporal sites following the sudden onset of a face stimulus (27). This component is particularly interesting since it differs reliably between faces and other stimuli in neurotypical individuals (see 28 for review) and reflects the interpretation of a stimulus as a face, beyond the physical characteristics of the visual input (29–31). An extensive amount of research has investigated how the N170 may be different in individuals with ASD versus TD controls. A recent meta-analysis pointed to a small but significant delay in N170 latency in response to faces in ASD compared to TD controls (32). However, the effect is not systematically found and does not relate to behavioral measures of social functioning in ASD (33). Moreover, its specificity is questionable, since it may reflect the generally slower processing of meaningful, even non-social, visual stimuli (34). Neural processing of social and nonsocial stimuli has also been studied through functional near-infrared spectroscopy (fNIRS). Atypicalities in the neural processing of social information in 4–6 month old infants at high familial risk for ASD were demonstrated (35) and replicated in an independent sample (36). While these methods provide information about the covert processing of social and nonsocial information, they are limited by the need to present social and nonsocial stimuli *at different times*, in order to isolate and compare neural responses to each of them.

To address this limitation, our recent study (37) relied on an EEG frequency-tagging approach [(38), see (39) for review] to investigate to what extent school-aged boys with and without ASD show a bias toward social stimuli. Specifically, we *simultaneously* presented two stimulation streams of widely varying images of faces or houses, tagged at different frequency rates, next to each other. With eye-tracking, we measured the fixations within specific areas of interest spanning each stimulus type, thereby offering an index of the overt attentional preference. With EEG, we measured the amplitude of the frequency-tagged electroencephalographic response to each of the stimulus types, thereby offering an index of the neural saliency of each type of stimuli. Frequency-tagged EEG showed enhanced neural responses for faces versus houses in the TD group, and a significant reduction of this social bias in boys with ASD as compared to TD boys. Importantly, this reduced social bias in ASD, as indexed by a group by stimulus type interaction, was already significant after only 5 s of stimulus presentation. Frequency-tagging EEG responses and eye tracking results (i.e., proportional looking times) were highly correlated, implying that individuals who looked relatively more at the stream of faces also showed higher face-tagged EEG responses. However, solely based on the eye-tracking results, we could not conclude that social preference was significantly reduced in the ASD group. Thus, the eye-tracking preferential looking data did not differentiate significantly between both groups, whereas the frequency-tagging EEG data did. Moreover, and unfortunately, participants looked in between both streams of stimuli for a large proportion of time. Another issue is that individual differences in spatial attention and attentional disengagement might also have affected the amplitude of the neural responses, and individuals with ASD have been reported to present alterations in both these domains. Indeed, orienting to a visual stimulus outside the current focus of attention requires two (potentially separable) components: First, one must disengage from whatever currently occupies one’s attention, and, second, one must shift to the peripheral stimulus (40, 41). Pertaining to visuo-spatial attention, individuals with ASD have been reported to present a sharper focus of attention (42) and they may benefit less and more slowly from a spatial cue in a Posner task (43). Pertaining to attentional disengagement, and in line with the restricted and repetitive behaviors and the characteristic difficulties in flexibility in ASD, a systematic review (41) concluded that there is robust behavioral and electrophysiological evidence from infants, children, and adults that autistic individuals have difficulties with disengagement. Mo *et al*. (44) further showed that this difficulty with attentional disengagement is rather domain-general and not specific to social stimuli.

**Figure 1:**
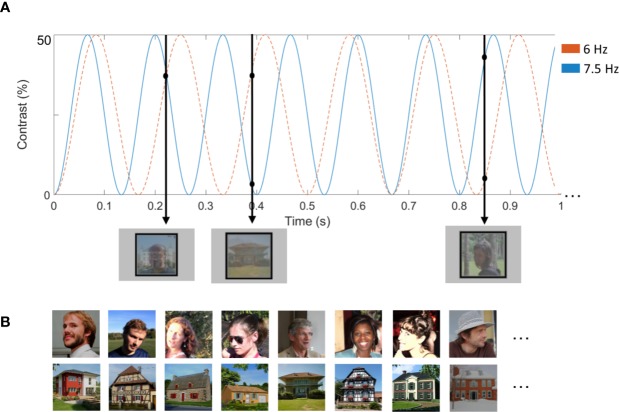
**(A)** Illustration of a stimulation sequence. The total experiment consisted of four sequences of 60 s. We counterbalanced frequencies (6 and 7.5 Hz) of the stimuli. In the illustrated example, images of houses were presented at 6 Hz, while images of faces were presented at 7.5 Hz. In the other two trials, faces were presented at 6 Hz and houses at 7.5 Hz. Images were contrast-modulated from 0 to 50%. The first black arrow depicts what was presented at 0.22 s. At this time point, the second face is presented at approximately 30% contrast, while the second house is also presented at 30% contrast. **(B)** Examples of face and house stimuli. Written informed consent was obtained from the individuals for the publication of the images.

Based on these considerations, the present study aims at improving our measures and strengthen our previous observations by spatially superimposing the two types of stimulus streams, so that differences in looking patterns, spatial attention, and disengagement cannot influence the processing saliency of each stimulus category. More precisely, while recording EEG signals, we present two streams of widely varying images of faces and houses, tagged at different frequency rates, simultaneously and superimposed at exactly the same position (**Figure 1**; **Movie S1**). Combining frequency-tagging with EEG allows disentangling neural responses to each of the stimulation streams, even when they are superimposed. Previous frequency-tagging EEG research with superimposed stimuli has shown that attention can modulate neural processing in a nonspatial manner. Enhanced processing (indicated by increased frequency-tagging EEG responses) of particular visual features (e.g., color, orientation, or direction of motion) or objects has been reported when those are attended, even when they are spatially overlapping [e.g., (45–49)]. In particular, one study presented spatially overlapping frequency-tagged face and house images while magnetoencephalography (MEG) responses were monitored as participants attended to the overlapping streams for cued targets. By combining the frequency-tagged MEG responses with functional ROIs defined from functional MRI (fMRI), the researchers found that attention to faces resulted in enhanced sensory responses in a face-selective region of the fusiform gyrus, whereas attention to houses resulted in increased responses in a place-selective region of the parahippocampal gyrus (50).

In the current study, images of natural faces (highly varying across viewpoint, luminance etc …) were used as a prototype of the social category. Pictures of houses were used as the non-social category, as often used in neuroimaging and electrophysiology research to compare to faces, including recent studies in ASD (33). Pictures of houses are typically associated with responses in medial regions of the ventral occipitotemporal cortex, such as the collateral sulcus and the parahippocampal gyrus (50–54) whereas faces typically elicit responses in the lateral parts of the middle fusiform gyrus (latFG) and in the inferior occipital gyrus (IOG) of the ventral occipito-temporal cortex (VOTC) (55–58) [see (59) for a direct comparison using human intracerebral recording data]. Moreover, faces and houses evoke quantitatively and qualitatively different category-selective responses in scalp EEG (60).

In general, we expect to find a strong social bias in TD children, as indicated by larger frequency-tagged EEG amplitudes in response to face stimuli as compared to house stimuli. Based on the literature and in accordance with our previous study (37), we expect that children with ASD will show a reduced social bias compared to TD children, or even that the social bias may be absent.

## Material and Methods

### Participants

We recruited 47 boys, aged 8-to-12 years old. To match the groups on verbal and performance IQ (VIQ, PIQ) five participants (two from the TD group, three from the ASD group) were *a priori* excluded from the reported analyses, resulting in a sample of 21 typically developing (TD) boys (mean age = 11.0 years ± SD = 1.2) and 21 boys with ASD (mean age = 10.9 ± 1.5, **Table 1**). However, inclusion of these participants did not change any results of the analyses. The sample in this study is identical to the one in the previous study (37), where social and non-social stimuli were presented side-by-side. All participants had normal or corrected-to-normal vision, and had a verbal and performance IQ above 80. Thirty-nine participants were right-handed. Participants with ASD were recruited through the Autism Expertise Center of the University Hospitals Leuven, Belgium. TD participants were recruited through elementary schools and sports clubs.

**Table 1:** Participant characteristics.

	ASD (mean ± SD)	TD (mean ± SD)	*t(df)*	*p*
Verbal IQ	107 ± 12	112 ± 12	t(40) = −1.41	0.18
Performance IQ	104 ± 15	110 ± 14	t(40) = −1.44	0.21
Age	10.8 ± 1.6	11 ± 1.2	t(40) = 0.80	0.43
Social Responsiveness Scale (*T*-score)	85 ± 12	42 ± 6	t(40) = 14.57	<.0001

Participant exclusion criteria were the presence or suspicion of a psychiatric, neurological, learning, or developmental disorder [other than ASD or comorbid attention deficit hyperactivity disorder (ADHD) in ASD participants] in the participant or in a first- or second-degree relative. This was assessed with a checklist filled out by the parents. Inclusion criteria for the ASD group were a formal diagnosis of ASD made by a multidisciplinary team in a standardized way according to DSM-IV-TR or DSM-5 criteria (1) and a total *T*-score above 60 on the Social Responsiveness Scale [SRS parent version (61)]. Seven participants with ASD took medication to reduce symptoms related to ASD and/or ADHD (Rilatine, Concerta, Aripiprazol). The TD sample comprised healthy volunteers, matched on age, verbal and performance IQ. Parents of the TD children also completed the SRS questionnaire to exclude the presence of substantial ASD symptoms. Descriptive statistics for both groups are displayed in **Table 1**, showing that they did not differ for age and IQ. Evidently, both groups differed highly significantly on SRS scores.

The Medical Ethical Committee of the university hospital approved the study, and the participants as well as their parents provided informed consent according to the Declaration of Helsinki. All participants received a monetary reward and a small present of their choice. The experiment was embedded in a larger research project consisting of three testing sessions. Intellectual abilities were assessed in a separate session. The current frequency-tagging experiment was included in the third session.

### IQ Measures

An abbreviated version of the Dutch Wechsler Intelligence Scale for Children, Third Edition [WISC-III-NL; (62, 63)] was administered. Performance IQ was estimated by the subtests Block Design and Picture Completion, verbal IQ by the subtests Vocabulary and Similarities (64).

### Frequency Tagging Experiment

#### Stimuli

Forty-eight color images of faces and 48 images of houses were used, all within their original background, making the images widely variable. Stimuli were selected from (65) and (60). Amplitude spectra of the face and house stimuli are available in supplementary material (**Figure S1** and **Figure S2, Supplementary Material**). The spectral analyses show that house stimuli have more energy in higher spatial frequencies and cardinal orientations. Faces and houses were presented superimposed on the screen, with a broad rectangular outline around them (**Figure 1**): one stimulation stream presented faces, and the other stream presented houses. All images differed highly in terms of viewpoint, lighting conditions and background. All stimuli were resized to 250 x 250 pixels, had equal pixel luminance and root-mean-square contrast on the whole image. Shown at a distance of 60 cm, and at a resolution of 1,920 x 1,200, the stimuli subtended approximately 13° of visual angle. Both the face and the object images were presented in a random order.

#### Procedure

After electrode-cap placement, participants were seated at a viewing distance of 60 cm and were instructed to maintain a constant distance. Stimuli were displayed on the screen [24-in. light-emitting diode (LED)-backlit liquid crystal display (LCD) monitor] through sinusoidal contrast modulation on a light grey background using Java. We used a screen with a refresh rate of 60 Hz, ensuring that the refresh rate was an integer multiple of the presentation frequencies. A sequence lasted 64 s, including 60 s of stimulation at full contrast, flanked by 2 s of fade-in and fade-out, with contrast gradually increasing and decreasing between 0 and 50%. Fade-in and fade-out were used to avoid abrupt eye movements and eye blinks due to the sudden appearance or disappearance of flickering stimuli. In total, there were four sequences, hence the total duration of the stimulus presentation was about 4 minutes.

**Figure 1** and **Movie S1** (**Supplementary Material**) illustrate a sequence, consisting of two streams of simultaneously presented series of images. In each sequence, images of one stimulus category were presented at 6 Hz and images of the other category at 7.5 Hz. The two streams of images were superimposed to one another and shown at the center of the screen. All images were drawn randomly from their respective categories, cycling through all available images before any image repetition. The presentation rate (6 *vs.* 7.5 Hz) was counterbalanced across both stimulus types (faces *vs.* houses), resulting in two conditions presented in a randomized order. The presentation frequencies were selected so that they are close to each other, in order to minimize differences in absolute EEG response (39, 66, 67).

Participants were instructed to look freely at the images on the screen and to press a key whenever they detected brief (300 ms) changes in the color of the rectangular outline surrounding the images. These color changes occurred randomly, 15 times per sequence. This task was orthogonal to the effect/manipulation of interest and ensured that participants maintained a constant level of attention throughout the entire experiment.

### Electroencephalography Recording

EEG was recorded using a BioSemi ActiveTwo amplifier system with 64 Ag/AgCl electrodes. During recording, the system uses two additional electrodes for reference and ground (CMS, common mode sense, and DRL, driven right leg). Horizontal and vertical eye movements were recorded using four electrodes placed at the outer canthi of the eyes and above and below the right orbit. The EEG was sampled at 512 Hz.

### Electroencephalography Analysis

#### Preprocessing

All EEG processing was performed using Letswave 6 (https://www.letswave.org/) and MATLAB 2017 (the MathWorks). EEG data was segmented in 67-s segments (2s before and 5s after each sequence), bandpass filtered (0.1 to 100 Hz) using a fourth-order Butterworth filter, and downsampled to 256 Hz. Next, noisy electrodes were linearly interpolated from the three spatially nearest electrodes (not more than 5% of the electrodes, -i.e., three electrodes, were interpolated). All data segments were re-referenced to a common average reference. While in frequency-tagging studies we typically apply blink correction (using ICA) for any participant blinking more than 2 SD above the mean [e.g., (68–70)], in the present study we did not perform any blink correction as none of the participants blinked excessively, i.e., more than two standard deviations above the mean across all participants (0.36 times per second). Note that frequency-tagging yields responses with a high SNR at specific frequency bins, while blink artefacts are broadband and thus do not generally interfere with the responses at the predefined frequency (67). Hence, blink correction (or removal of trials with many blinks) is not systematically performed in such studies [e.g., (71–73)].

#### Frequency-Domain Analysis

Preprocessed segments were further cropped to contain an integer number of 1.5 Hz cycles (i.e., largest common divisor of both 6 and 7.5 Hz), beginning after fade-in and until 59.38 s (15,203 time bins). The resulting segments were averaged per condition (i.e., segments with the same combination of stimulus category and presentation rate) in the time domain to preserve the complex phase of the response and reduce EEG activity out-of-phase with the stimulation (i.e., noise). The averaged waveforms were transformed into the frequency domain using a Fast Fourier transform (FFT), and the amplitude spectrum was computed with a high spectral resolution (0.017 Hz, 1/59.38 s) resulting in a very high signal-to-noise ratio (SNR) (39, 67).

**Figure 2:**
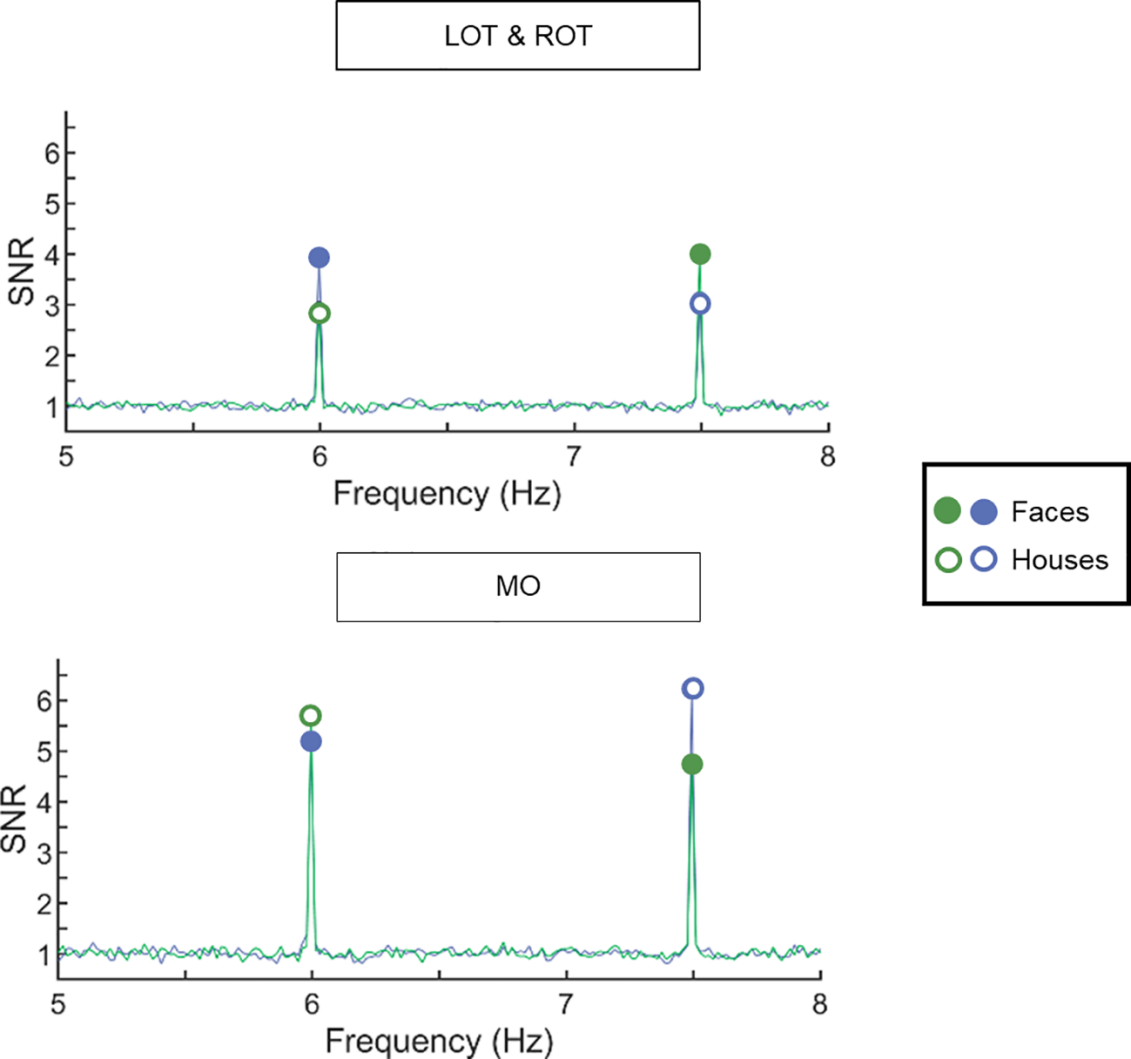
Signal-to-noise ratio (SNR) spectra averaged across all participants (across the two groups) show clear responses at the first harmonic frequencies of interest. Data are plotted for the left and right occipito-temporal region (upper panel) and the medial occipital region (lower panel). The frequency spectrum is plotted from 5 to 8 Hz. In green, images of houses are presented at 6 Hz, while images of faces were presented at 7.5 Hz. In blue, the frequencies were reversed. Full circles display the neural response for faces, empty circles display the neural response for houses. In left and right occipito-temporal ROIs, the response to faces is larger than to houses. In the medial occipital ROI, the response to houses is larger than the response to faces.

**Figure 3:**
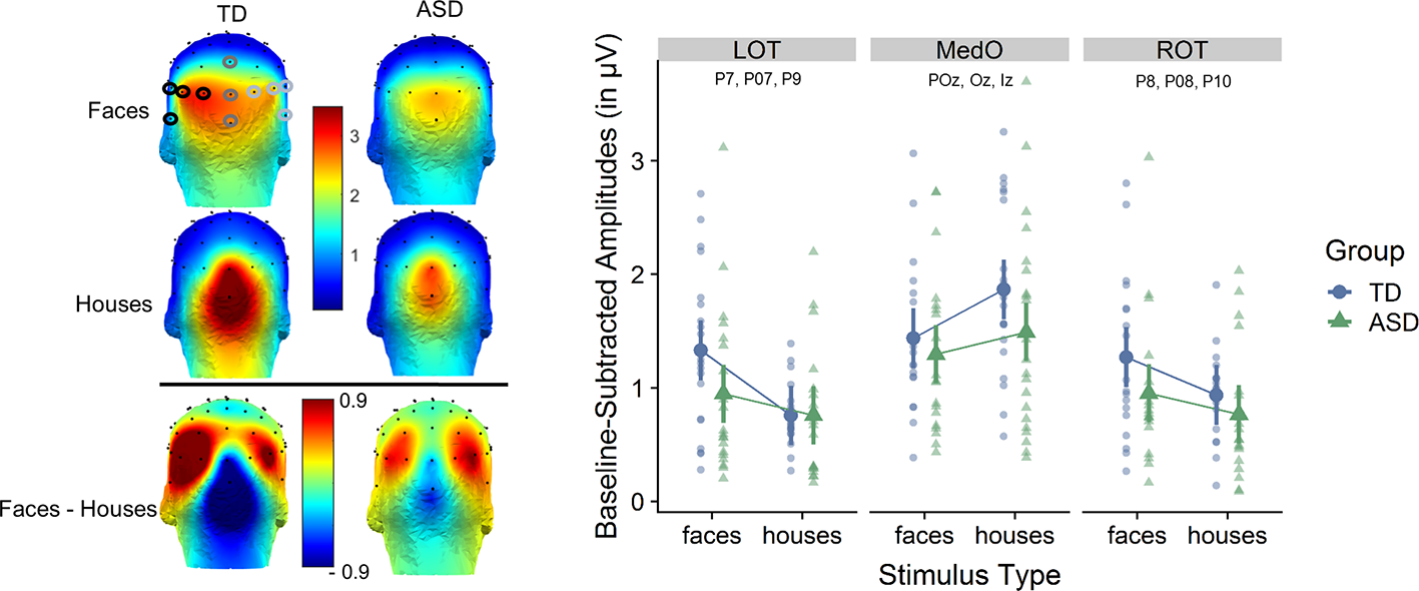
Left: scalp distribution of the electroencephalography (EEG) signal during fast periodic visual stimulation (baseline subtracted amplitudes in µV). Frequency-tagged neural responses to the streams of periodically presented faces and houses are shown for each participant group, as well as the differential response for faces minus houses. The analysis of the response to both types of stimuli focused on three regions of interests (ROIs): medial occipital (MO: Iz, Oz, POz), left occipito-temporal (LOT: O1, PO7, P7, P9), and right occipito-temporal (ROT: O2, PO8, P8, P10). Right: averaged baseline-subtracted amplitudes for each stimulus condition (faces or houses) for each group and for each ROI. The individual subject data is displayed in the background. Statistical analysis shows an interaction between group, stimulus type and ROI.

The recorded EEG contains signal at harmonics frequencies (i.e., integer multiples) of the frequencies at which images are presented (6 and 7.5 Hz) (39, 67). We used two measures to describe the response in relation to the noise level: signal-to-noise ratio (SNR) to better visualize the data [e.g., (74)] and baseline-corrected amplitudes to quantify the response across harmonics (65). SNR spectra were computed for each electrode by dividing the value at each frequency bin by the average value of the 20 neighboring frequency bins (12 bins on each side, i.e., 24 bins, but excluding the 2 bins directly adjacent and the 2 bins with the most extreme values). **Figure 2** displays the SNR spectra. We computed baseline-corrected amplitudes in a similar way by subtracting the average amplitude of the 20 surrounding bins. For group visualization of topographical maps (**Figure 3**), we computed across-subjects averages of the baseline-corrected amplitudes for each condition and electrode separately.

Since the response is inherently distributed over multiple harmonics and all the harmonic frequencies represent some aspect of the periodic response, we combine the response amplitudes across all those harmonics whose response amplitude is significantly higher than the amplitude of the surrounding noise bins [as recommended in (65)]. To define the harmonics that were significantly above noise level, we computed Z-score spectra on group-level data for each stimulation frequency (60, 68, 74, 75). We averaged the FFT amplitude spectra across electrodes in the relevant regions-of-interest (ROIs) based on topographical maps, and transformed these values into Z-scores (i.e., the difference between the amplitude at each frequency bin and the mean amplitude of the corresponding 20 surrounding bins, divided by the SD of the amplitudes in these 20 surrounding bins). For 6 Hz, Z-scores were significant (i.e., *Z* > 2.32 or *p* < 0.01) until the 5^th^ harmonic (30 Hz) and for 7.5 Hz, Z-scores until the fourth harmonic (30 Hz) were significant. To include an equal number of harmonics for both stimulation frequencies and to exclude shared harmonics (30 Hz), we selected the first three harmonics for both frequencies and summed the baseline-corrected amplitudes of those for each frequency and each condition separately. Hence, we quantified neural responses to faces and houses at 6 Hz and at 7.5 Hz by summing the baseline-subtracted responses for 3 harmonics: 6, 12, and 18 Hz for the 6 Hz stimulation frequency; and 7.5, 15, and 22.5 Hz for the 7.5 Hz stimulation frequency. Therefore, we obtained an index of neural saliency per stimulus type (i.e., houses versus faces) and per presentation rate.

Based on *a priori* knowledge, in accordance with previous studies and confirmed by visual inspection of the topographical maps of both groups (**Figure 3**), we identified regions of interest (ROI) in which the signal was maximal and averaged the signal at these nearby electrodes. The analysis of the response to both types of stimuli focused on three ROIs: medial occipital (MO: Iz, Oz, POz), left occipito-temporal (LOT: O1, PO7, P7, P9) and right occipito-temporal (ROT: O2, PO8, P8, P10) (**Figure 3**).

#### Statistical Analysis

We statistically analyzed the baseline-corrected amplitudes in each ROI and at each presentation frequency for each stimulus type at the group-level using general linear mixed-effects models (LMEMs) using the AFEX package v0.22-1 (76) in R v3.4.3 (R Core Team, 2012). In particular, we examined the neural responses (i.e., baseline-subtracted amplitudes) with *stimulus type* (houses *vs.* faces) and *ROI* (MO, LOT, ROT) as within-subject factors, and *group* (ASD *vs.* TD) as a between-subject factor. We included a random intercept per participant in the model. *Post-hoc* T-tests were performed on the fitted model using the emmeans package (77). Tukey-corrected p-values were used to compare means and unstandardized effect sizes are reported [cf. (78, 79)].

In addition, we determined the significance of responses for each individual participant and each stimulus type as follows [e.g., (66, 69, 71)]: 1) the raw FFT amplitude spectrum was averaged across electrodes per ROI, and 2) cut into segments centered on the target frequency bin and harmonics (i.e., 6, 12, 18 Hz or 7.5, 15, 22.5 Hz), surrounded by 20 neighboring bins on each side; 3) the amplitude values across the segments of FFT spectra were summed; 4) the summed FFT spectrum was transformed into a *z*-score using the 20 surrounding bins (see above). Responses of a given participant were considered significant if the z-score at the target frequency bin exceeded 1.64 (i.e., *p* < 0.05 one-tailed: signal > noise). Finally, we computed spearman correlations between the neural measures and the scores on the Social Responsiveness Scale (SRS). To this end, we used the corrplot package in R (78).

## Results

### No Group Difference in Orthogonal Task Performance

Both groups performed equally on the behavioral color change detection task, suggesting a similar level of attention throughout the experiments. Both groups showed accuracies between 97 (SD = 6%) and 97.1% (SD = 3.9%) with mean response times between 0.47 (SD = 0.07) and 0.46 (SD = 0.04) seconds, for ASD and TD respectively. Statistical analyses (two-sided t-tests) showed no significant differences between the ASD group and the TD group [accuracy: t(36) = -0.03, p = 0.49; response times t(36)= 0.71, p = 0.24].

### Electroencephalography Responses in Autism Spectrum Disorder Participants Are Not Modulated by Social Versus Non-Social Stimulation

We observed robust frequency-tagged responses, in the three regions of interest (ROI) and for the two stimulus types (see **Figure 2** for SNR spectrograms and **Figure 3** for scalp distributions and averaged response amplitudes). Analyses at the individual level indicated that, despite the short recording time, all participants showed significant responses to houses and to faces in the pre-specified ROIs.

At the group level, statistical analyses showed a main effect of *stimulus type* [*F*(1,441) = 5.02; *p* = 0.026] [faces (1.20 µV) larger than houses (1.08 µV) and a main effect of *ROI* (F(2,441)= 58.10, *p <*0.0001] [larger responses in MO (1.51 µV) than in LOT (0.94 µV) and ROT (0.97 µV)]. These effects were qualified by a significant interaction effect between *stimulus type* and *ROI* [*F*(2,441) = 19.10, *p* < 0.0001] and, most importantly a significant three-way interaction between *group*, *stimulus type*, and *ROI* [*F*(2,441) = 3.40, *p* = 0.034)]. *Post-hoc* testing revealed that over the left occipito-temporal channels, the response for faces (1.32 µV) was larger than for houses (0.75 µV) in the TD group [*T*(441) = 4.73, *p* = 0.0002)]. While over the ROT channels the responses were also higher to faces (1.26 µV) than to houses (0.91 µV) in the TD group, this effect did not reach significance [*T*(441) = 2.75, *p* = 0.207]. Over medial occipital channels, responses to houses (1.82 µV) were significantly higher than to faces (1.44 µV) in the TD group [*T*(441) = −3.54, *p* = 0.0226]. In contrast, in the ASD group, the responses to faces were not significantly different from responses to houses, in none of the ROIs [LOT: *T*(441) = 1.6, *p* = 0.91, ROT: *T*(441) = 1.56, *p* = 0.92, MO: T(441) = −1.64, *p* = 0.89)]. Mean amplitude values for the ASD group in LOT were 0.95 µV (faces) and 0.76 µV (houses); in ROT 0.95 µV (faces) and 0.77 µV (houses); and in MO 1.30 µV (faces) and 1.49 µV (houses).

Taken together, the three-way interaction indicates that the neural organization of the TD participants is more differentiated and specialized in terms of anatomically localized stimulus-specific responses, whereas the response pattern of the ASD group is not modulated by the social versus non-social character of the stimulation.

**Figure 4:**
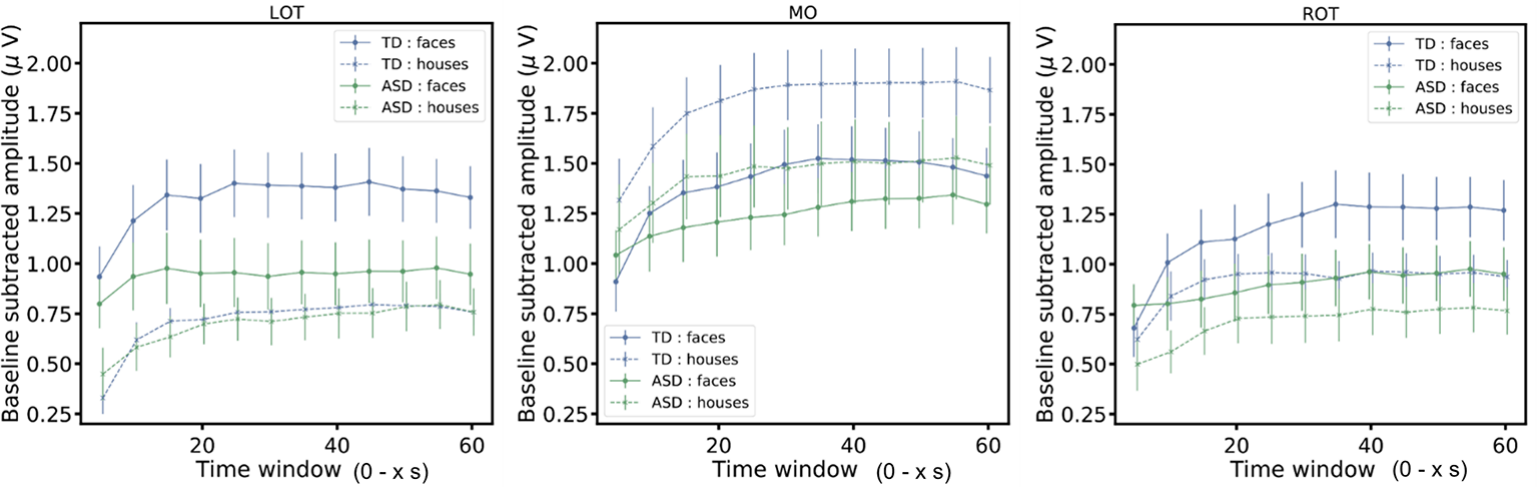
The figure shows the baseline subtracted amplitudes of the responses for segments of increasing length (5 to 60 s in steps of 5 s: 5, 10, 15,…, 60 s). The mean amplitudes (± SEM) are displayed.

In addition, we considered how the neural responses evolved over the course of a stimulation sequence, as this could also inform about the minimal sequence length needed to observe an interaction effect. To do so, we cut the original data in segments of increasing length (5 to 60 s in steps of 5 s: 5, 10, 15,…, 60 s). For all segments, we plotted the evolution of the signal relative to the sequence duration (**Figure 4**). Overall, after an initial buildup period, mean amplitudes remain stable over the trial duration and reflect the findings described above. More specifically, in left and right OT channels, the *group* x *stimulus type* interaction (indicative of a differential social bias in ASD *vs.* TD) is significant from 25 s onwards [*F*(1,285) = 4.61, *p* = 0.03)] and remains significant during the entire trial (*p* < .05).

### No Associations Between Neural Responses and Social Responsivity

Spearman correlations showed that individual differences in the amplitude of the neural responses were not significantly related to individual differences in social responsivity as reported by the parents on the SRS questionnaire. Neither the difference between faces and houses, nor the amplitudes of faces and houses separately were correlated with the SRS. This was the case within the two groups and across the groups.

## Discussion

Individuals with autism spectrum disorder (ASD) have difficulties with social communication and interaction. Here, we quantified the saliency of processing social versus non-social information by frequency-tagging superimposed streams of widely variable images of faces and houses while recording EEG. This approach allows monitoring brain responses to simultaneously presented stimuli, and, importantly, changes in response amplitude represent dynamic neural changes related to the intensity of processing the driving stimulus. Whereas a recent study showed reduced social bias in ASD using a frequency-tagging EEG approach with streams of social and non-social stimuli presented side-by-side (37), here we extend and specify these findings by presenting the stimulation streams superimposed. By doing so, we can specifically measure the neural processing and saliency of each stimulus category, while ruling out potential confounds related to looking patterns, spatial attention and attentional disengagement.

Within a short amount of time (i.e., four trials of 60 s), we observed significant responses for each participant and each stimulus type. These responses were implicit in the sense that they did not require any active behavior of the participant, apart from looking at the screen. Importantly, they were determined in an objective manner since they were locked to the stimulation frequencies (39, 67) and did not require any subjective interpretation on the part of the researcher. The stimulation-tagged brain responses were located over medial occipital and occipito-temporal regions. Results showed a significant interaction between stimulus type, group and regions of interest (ROI). In the TD group, faces elicited larger responses than houses over occipito-temporal channels, while houses evoked stronger responses than faces over medial occipital channels. Conversely, in the ASD group, the differences between faces and houses were not significant in any of the ROIs. In other words, TD participants showed a differentiated localization and tuning of the neural responses toward social versus non-social stimuli, whereas the response pattern of the ASD group was not modulated by the social versus non-social character of the stimulation.

Reduced interest in social stimuli in ASD might result in less frequent engagement with faces. Accordingly, developing neural systems devoted to face processing may lack experience-expectant visual input, which may be necessary for establishing the neural architecture for expert face processing competency (2). Here, we show that even when individuals with ASD show similar spatial attention to the stream of faces, EEG frequency-tagging still evokes lower face-selective neural activity in occipito-temporal areas as compared to TD individuals. We observed that in the TD group, faces elicited larger responses than houses over occipito-temporal channels, while houses evoked stronger responses than faces over medial occipital channels. This result is in line with previous observations, indicating that lateral ventral occipito-temporal brain regions (i.e., inferior occipital gyrus, lateral fusiform gyrus) respond preferentially to face stimuli while medial occipito-temporal structures (medial temporal gyrus, collateral sulcus, and parahypocampal gyrus) display a preference for house stimuli (51, 53, 54, 80). Likewise, previous research combining frequency-tagging MEG with functional ROIs defined from fMRI showed that attention allocation selectively modulated the amplitude of the frequency-tagged responses to superimposed stimuli: attention to faces resulted in selectively enhanced responses in the fusiform area, whereas attention to houses increased the neural responses in the parahippocampal place area (50). In addition, the medial occipital brain topography in response to the houses may be particularly driven by particular low-level characteristics of the houses, such as rectangular features (81) and cardinal orientation (82, 83). Indeed, in general, houses have more energy in higher spatial frequencies and cardinal orientation, as was also confirmed by the amplitude spectra of the face and house stimuli used in this study (**Figure S1, Supplementary Material**). Along these lines, previous ERP studies have shown larger amplitudes in early visual ERPs over medial occipital electrodes for images with more high spatial frequency content (84).

In the TD group, significantly increased responses to faces versus houses were found only in the left ROI. At first glance, this observation appears inconsistent with the typical right lateralization of the human cortical face network (57, 59, 85). Nevertheless, other studies in children within this age range have not found the right lateralization pattern for face preference that is typically observed in adults [e.g., (86)]. Moreover, studies using a frequency-tagging oddball EEG paradigm across different ages suggest a non-linear development of the right hemispheric specialization for human face perception (87). In 5 year old children (87) and 8–12 year old children (70), face-selective responses did not differ across hemispheres, while the same paradigm in adults [e.g., (76)] and in infants (88) elicits right lateralized electrophysiological occipito-temporal face-selective responses.

Strikingly, in the ASD group, the neural responses for faces and houses were not significantly different from each other in any of the ROIs. Previous observations already indicated altered sensitivity to face stimuli in the fusiform face area (FFA) of ASD (89–93), although this finding has not always been replicated (94–98). One possibility is that less frequent engagement with faces might have resulted in altered specialization of the FFA in ASD participants.

In a previous study (37), we presented streams of social and non-social stimuli side-by-side and we showed that frequency-tagging is a sensitive method allowing us to observe a reduced social bias in boys with ASD. Here, by superimposing both streams of stimuli, we showed that even in the absence of explicit looking behavior, frequency-tagging allows measuring the relative neural saliency of faces and houses. As a result, we quantified the implicit social bias in children with and without ASD, while controlling for potential influences of visuo-spatial attention and/or attentional disengagement. Against this background, our findings suggest that in children with ASD, as compared with TD children, the face-sensitive areas are less preferentially responsive for faces compared to houses and that the typical social bias in these areas is reduced. Our findings generally corroborate developmental accounts that relate social experiences to the specialized neural wiring of the face processing network.

Unexpectedly, however, children with ASD also show less differentiated responses to the house stimulation in the medial occipital region. Taken together, this suggests that, generally, the neural wiring in children with ASD is less differentiated and specialized, and less modulated by the social versus non-social character of the stimulation, which may possibly point toward a more general developmental delay in social and non-social visual sensory processing. This finding might echo broader predictive coding accounts of ASD, suggesting a generally atypical attentional information processing style that is manifested most clearly in the social domain—possibly to the high complexity inherent to social situations (13).

We did not observe significant correlations between the SRS and the face or house-related neural responses. The SRS measures the severity of ASD symptoms over a variety of domains, based on evaluations by the parents. Hence, while it gives a clear idea of the perceived symptoms in daily life, this measure does not purely reflect the actual behavior and performance, and is also determined by several other parent-related processes (e.g., whether there are other children in the family with an ASD diagnosis) (99). Second, the variation of amplitude of the EEG response across individuals also reflects general factors such as skull thickness and cortical folding (see the discussion in (100). While these factors should be neutralized when comparing relatively large groups of participants or when comparing different paradigms in the same participants, they add variance to amplitude differences within a group of individuals, reducing the significance of correlation measures [see (33, 101)]

Further studies are required to replicate this finding in a larger and more heterogeneous and representative sample. Given the short recording time of the frequency-tagging paradigm with multiple simultaneous inputs and the robustness of the individual responses, the method could be used as a fast marker of social preference in a wide range of populations, including low-functioning individuals with ASD, and young children and infants (87, 88). Therefore, the approach has potential to pinpoint developmental trajectories in longitudinal research, from infancy onwards. Moreover, implicit objective measures can help overcome the difficulty of interpreting behavioral findings (which may be influenced by many factors such as motivation, task understanding, etc.). Since in the current study no positional counterbalancing is needed, few trials are required in order to obtain robust data. This is especially an advantage when testing challenging populations, where testing time is limited.

## Data Availability Statement

The anonymized datasets generated for this study are available on request to the corresponding author.

## Ethics Statement

The studies involving human participants were reviewed and approved by Ethische commissie onderzoek UZ/KU Leuven. Written informed consent to participate in this study was provided by the participants’ legal guardian.

## Author Contributions

SVe, MD, SVa, BR, and BB conceived and designed the study. SVa and JS contributed to participant recruitment. SVe and SVa collected the data. SVe, CJ, and MD statistically analyzed and interpreted the data, and all authors discussed the results. SVe and BB drafted the manuscript. All authors provided feedback and approved the final version.

## Funding

This work was supported by grants of the Research Foundation Flanders (FWO; G0C7816N) and Excellence of Science (EOS) grant (G0E8718N; HUMVISCAT) and of the Marguerite-Marie Delacroix foundation.

## Conflict of Interest

The authors declare that the research was conducted in the absence of any commercial or financial relationships that could be construed as a potential conflict of interest.
