# Supplementary material for: Frequency-Tagging Electroencephalography of Superimposed Social and Non-Social Visual Stimulation Streams Reveals Reduced Saliency of Faces in Autism Spectrum Disorder
Source: Front Psychiatry. 2020 Apr 28;11:332. doi: 10.3389/fpsyt.2020.00332 (PMC7199527; doi:10.3389/fpsyt.2020.00332)
Supplement: Supplementary file 1 [file DataSheet_1.docx]

Supplemental information

**Stimulus analysis: Quantification of the amplitude spectrum difference between face and house images.**

Images of faces and houses are thought to differ in the rate of decrease of energy/contrast as a function of spatial frequency; this rate being around 1/frequency for objects and natural scenes and 1/frequency² for faces (Keil, 2008). We quantified this difference in amplitude spectrum (AS) between the used images of faces (48) and houses (48) as follows: (1) the AS of each image was summarized by computing the radial average of the AS across all orientations for each spatial frequency (Figure S1 A, B), (2) the resulting AS were converted to log-log scale and fitted with a linear function (Figure S1 C), (3) the slope of the line, computed for each image, was used to compare the reduction of contrast as a function of spatial frequency across faces and houses. These slopes were significantly higher for faces (*p* < 0.0001, 2-tailed permutation test), indicating lower contrast at higher spatial frequency (> ~15 Cy/image) compared to houses (Figure S1 C). Moreover, as shown in Figure S1A, houses had much more contrast at cardinal spatial frequency orientations (i.e. vertical and horizontal), while in images of faces, contrast was more homogeneous across spatial frequency orientations.


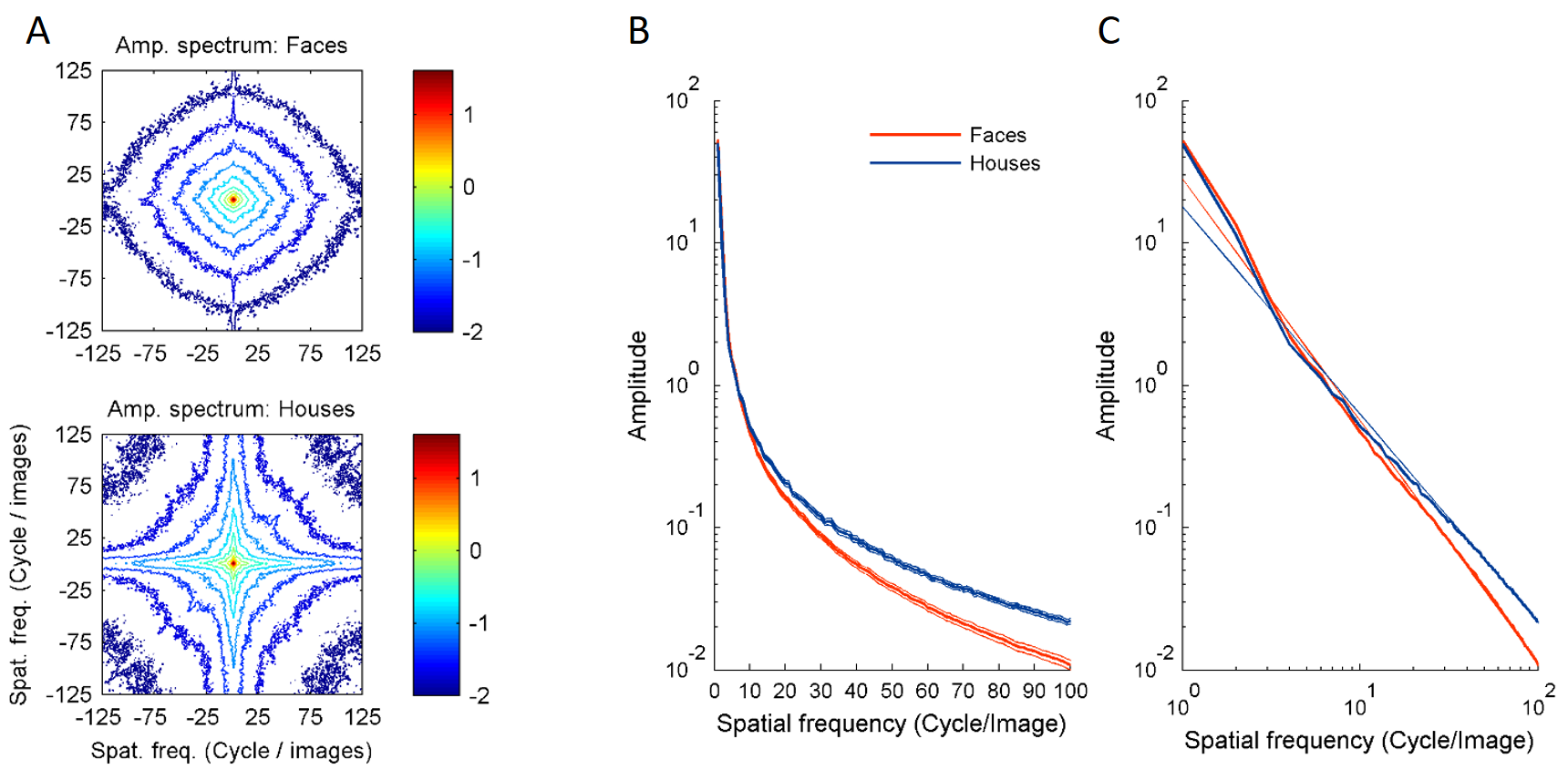


**Figure S1 : Amplitude spectrum difference between face and house images.** A. Contour plots showing the amplitude spectrum (AS) averaged across images of faces (top) and houses (bottom). B. Radial averages of the AS (i.e. average of amplitudes across all orientations computed for each spatial frequency - SF) for face and house images. Full line shows the mean, dotted line shows the standard error. Y-axis is in log scale. The plot shows the higher amplitude for houses for spatial frequencies above ~15 Cy/image. C. The slope of the AS (i.e. reduction of amplitude with increasing SF) is estimated for each image by fitting a linear function to the radial average of AS transformed to a log-log scale. The AS for faces and houses is shown as a thick line and the linear fit as a thin line.


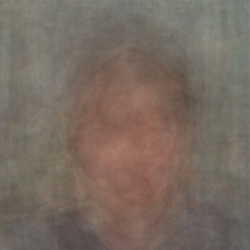

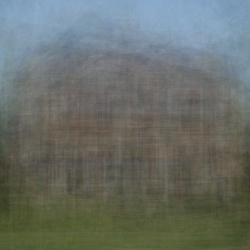


**Figure S2 : The average across all face and house images shows more vertical and horizontal high spatial frequency lines for houses.**

**Reference list**

Keil, M.S. (2008). Does face image statistics predict a preferred spatial frequency for human face processing? Proc. R. Soc. B Biol. Sci. *275*, 2095–2100.
